# From policy to plate: strengthening implementation of Canada’s national school food policy

**DOI:** 10.17269/s41997-025-01089-3

**Published:** 2025-07-18

**Authors:** Annette Blais, Benjamin Organ, Nicole Weber, Mavra Ahmed, Mary L’Abbé, Daniel Sellen, Vasanti Malik

**Affiliations:** 1 Department of Nutritional Sciences, Temerty Faculty of Medicine, University of Toronto, Toronto, ON Canada; 2 Joannah and Brian Lawson Centre for Child Nutrition, University of Toronto, Toronto, ON Canada; 3 Dalla Lana School of Public Health, University of Toronto, Toronto, ON Canada; 4 Department of Anthropology, Faculty of Arts & Science, University of Toronto, Toronto, ON Canada; 5 Department of Nutrition, Harvard T.H. Chan School of Public Health, Harvard University, Boston, MA USA

**Keywords:** National school food policy, Canada, School food program, Funding, Nutrition guidelines, Policy evaluation, Politique nationale d’alimentation scolaire, Canada, Programme d’alimentation scolaire, Financement, Directives nutritionnelles, Évaluation des politiques

## Abstract

Canada’s newly released National School Food Policy (NSFP) and its associated federal funding commitments mark significant progress toward a more coordinated school food system in Canada. However, significant gaps remain that could undermine the policy’s effectiveness and longevity. Current bilateral agreements between federal and provincial/territorial governments highlight disparities in per-student funding, raising concerns about whether the allocated resources are adequate to achieve universally accessible, high-quality meal provision. Additionally, while the NSFP encourages school food programs to adhere to Canada’s Food Guide and provincial nutrition guidelines, ambiguous language and the absence of enforceable standards leave room for inconsistent nutritional quality across programs. Furthermore, without a structured framework for monitoring and evaluation, there is no mechanism to assess program effectiveness, track nutritional outcomes, or ensure program accountability. To maximize the NSFP’s impact, we urge policymakers to establish transparent funding rationales, develop clear and evidence-based national guidelines, and implement robust monitoring systems. Strengthening these policy components is essential to ensuring an equitable, effective, and sustainable approach to NSFP implementation in Canada.

## Résumé

La politique nationale d’alimentation scolaire (PNAS) récemment publiée par le gouvernement fédéral du Canada, et les engagements financiers qui y sont associés, marquent un progrès significatif vers un système d’alimentation scolaire mieux coordonné au Canada. Toutefois, il subsiste des déficits importants qui pourraient nuire à l’efficacité et à la longévité de cette politique. Les accords bilatéraux actuels entre le gouvernement fédéral et les gouvernements provinciaux et territoriaux mettent en évidence des disparités dans le financement par élève, ce qui soulève la question de savoir si les ressources allouées sont suffisantes pour assurer l’accès universel à des repas de qualité. En outre, bien que la PNAS encourage les programmes d’alimentation scolaires à adhérer au Guide alimentaire canadien et aux lignes directrices provinciales en matière de nutrition, le langage ambigu et l’absence de normes applicables laissent place à une qualité nutritionnelle inégale d’un programme à l’autre. De plus, en l’absence d’un cadre structuré de suivi et d’évaluation, il n’existe aucun mécanisme permettant d’évaluer l’efficacité du programme, de suivre les résultats nutritionnels ou d’assurer la responsabilité du programme. Pour maximiser l’impact de la PNAS, nous faisons appel au gouvernement fédéral pour l’établissement de principes de financement transparents, de lignes directrices nationales claires et fondées sur des preuves, et de systèmes de suivi solides. Le renforcement de ces composantes politiques est essentiel pour garantir une approche équitable, efficace et durable de la mise en œuvre de la PNAS au Canada.

## Background

School food programs (SFPs) are well-established as important drivers of child nutrition, health, and academic achievement (Colley et al., 2019). However, Canada’s landscape of SFPs remains an ad hoc combination of provincial, municipal, and community-led initiatives that vary widely in structure, reach, and funding (Blais et al., 2024). Some programs are well-funded with capacity and resources to deliver high-quality meals, but most struggle with limited funds and rely on community-based support, leaving gaps in access and nutritional quality (Blais et al., 2024, 2025).

Following public, academic, and political interest in school food, Canada’s federal government committed $1 billion over 5 years in their 2024 budget to harmonize and expand SFPs nationwide (Prime Minister of Canada, 2024). Nevertheless, partisan divides persist regarding school feeding. While all non-Conservative Members of Parliament (MPs) (*n* = 204) supported Bill C-322, which sought to establish a national school food framework, all Conservative MPs (*n* = 110) opposed it (House of Commons Canada, 2023). Despite this, Bill C-322 passed, prompting the release of Canada’s first-ever National School Food Policy (NSFP), which outlines guiding principles and program objectives (National School Food Policy, 2024). Subsequently, the federal government introduced a $20.2 million School Food Infrastructure Fund (SFIF), targeted at not-for-profit organizations to support community-based SFPs through the purchase and installation of equipment and infrastructure (Prime Minister of Canada, 2024). Additionally, bilateral funding agreements were signed with all provinces and territories to advance SFPs (Table 1).

**Table 1:** Summary of national school food policy bilateral agreements by province and territory

Province/territory	Amount of federal funding disbursed over 3 years	Projected impact of federal funding	Release date of bilateral agreement	Provincial political party in power at time of signing
Newfoundland and Labrador^1^	$9.1 million	Feed an additional 4100 students	September 4, 2024	Liberal Party
Manitoba^2^	$17.2 million	Feed an additional 19,000 students	October 18, 2024	NDP
Ontario^3^	$108.5 million	Feed an additional 160,000 students	November 22, 2024	PC Party
Prince Edward Island^4^	$7.1 million	Provide an additional 1500 students with lunches and provide an additional 800 students with breakfasts and snacks	November 29, 2024	PC Party
New Brunswick^5^	$11.2 million	Feed an additional 57,000 students	February 7, 2025	PC Party
Nova Scotia^6^	$12.4 million	Enhance school breakfast programs	February 24, 2025	PC Association
Nunavut^7^	$7.6 million	Enhance existing school food programs	February 28, 2025	N/A: Consensus Government
Northwest Territories^8^	$7.4 million	Enhance existing school food programs for 8615 students	March 6, 2025	N/A: Consensus Government
Quebec^9^	$65.2 million	Support and improve school food services in Quebec, while providing more students in Quebec with meals and snacks at school	March 7, 2025	Coalition Avenir Québec
British Columbia^10^	$39.4 million	Enhance school food programs for 90,000 students	March 7, 2025	NDP
Yukon^11^	$7.4 million	Enhance existing school food programs	March 10, 2025	N/A: Consensus Government
Saskatchewan^12^	$15.8 million	Enhance and expand school food programs	March 10, 2025	Saskatchewan Party
Alberta^13^	$42.2 million	Enhance and expand school food programs	March 10, 2025	United Conservative Party

*NDP*, New Democratic Party; *PC*, Progressive Conservative. This table presents a summary of all bilateral school feeding agreements between the federal and provincial/territorial governments, including the total amount of funding to be disbursed over 3 years, the projected impact of federal funding (as reported in documentation from the federal government), the bilateral agreement release date, and the provincial political party in power at the time of agreement release. Provinces are ordered by date of bilateral agreement (oldest to most recent). Canada’s territories (Nunavut, Northwest Territories, Yukon) operate by consensus government; there are no political parties

^1^Newfoundland and Labrador Executive Council of Education. (2024, September 4). Healthy meals for kids in Newfoundland and Labrador. News Releases. https://www.gov.nl.ca/releases/2024/exec/0904n04/

^2^Government of Manitoba. (2024, October 18). Governments of Canada and Manitoba announce healthy meals for Kids in Manitoba. News Releases. https://news.gov.mb.ca/news/index.html?item=65619

^3^Employment and Social Development Canada. (2024, December 4). Making school meals a reality for more kids in Ottawa. Government of Canada. https://www.canada.ca/en/employment-social-development/news/2024/12/making-school-meals-a-reality-for-more-kids-in-ottawa.html

^4^Brun, S. (2024, November 29). Ottawa pledges $7 M to help expand P.E.I.’s School Food Program. CBC News. https://www.cbc.ca/news/canada/prince-edward-island/pei-school-food-program-federal-funding-1.7396627

^5^Employment and Social Development Canada. (2025, February 7). Healthy meals for kids, savings for families in New Brunswick. Cision Canada. https://www.newswire.ca/news-releases/healthy-meals-for-kids-savings-for-families-in-new-brunswick-815561775.html

^6^Employment and Social Development Canada. (2025, February 24). Healthy meals for kids, savings for families in Nova Scotia. Government of Canada. https://www.canada.ca/en/employment-social-development/news/2025/02/healthy-meals-for-kids-savings-for-families-in-nova-scotia.html

^7^Employment and Social Development Canada. (2025, February 28). More healthy meals for kids in Nunavut. Government of Canada. https://www.canada.ca/en/employment-social-development/news/2025/02/more-healthy-meals-for-kids-in-nunavut.html

^8^Employment and Social Development Canada. (2025, March 6). Healthy meals for kids, savings for families in the Northwest Territories. Government of Canada. https://www.canada.ca/en/employment-social-development/news/2025/03/healthy-meals-for-kids-savings-for-families-in-the-northwest-territories.html

^9^Employment and Social Development Canada. (2025, March 7). Healthy meals for students in Quebec. Government of Canada. https://www.canada.ca/en/employment-social-development/news/2025/03/healthy-meals-for-students-in-quebec.html

^10^Employment and Social Development Canada. (2025e, March 7). Healthy meals for kids, savings for families in British Columbia. Government of Canada. https://www.canada.ca/en/employment-social-development/news/2025/03/healthy-meals-for-kids-savings-for-families-in-british-columbia.html

^11^Employment and Social Development Canada. (2025, March 10). More healthy meals for kids in the Yukon. Government of Canada. https://www.canada.ca/en/employment-social-development/news/2025/03/more-healthy-meals-for-kids-in-the-yukon.html

^12^Employment and Social Development Canada. (2025, March 10). Healthy meals for kids, savings for families in Saskatchewan. Government of Canada.https://www.canada.ca/en/employment-social-development/news/2025/03/healthy-meals-for-kids-savings-for-families-in-saskatchewan.html

^13^Employment and Social Development Canada. (2025, March 10). Healthy meals for kids, savings for families in Alberta. Government of Canada. https://www.canada.ca/en/employment-social-development/news/2025/03/healthy-meals-for-kids-savings-for-families-in-alberta.html

While these developments mark critical progress, and there is a growing consensus around the need for a nationally harmonized school food system, gaps remain in implementation and accountability. This commentary highlights three foundational challenges threatening the NSFP’s ability to deliver equitable, high-quality meals and ensure long-term sustainability: funding sufficiency, the absence of clear and consistently adopted nutritional standards, and a lack of robust monitoring mechanisms. Each can be addressed by applying existing and gathering new knowledge for decision-making.

## Funding sufficiency

Ensuring adequate funding is essential for the success of the NSFP. Although the federal government’s commitment is positive, it remains unclear whether the allocated funding is sufficient to meet national goals. For example, Newfoundland and Labrador’s bilateral agreement of $9.1 million over 3 years aiming to feed an additional 4100 students (Prime Minister of Canada, 2024) amounts to approximately $740 per student annually, or $4 per student per school day. However, if the same funding were distributed across the province’s entire student population of 63,718 (Newfoundland & Labrador, 2024), it would equate to only $48 per student annually—less than $0.26 per student per school day. When considering the province’s existing $3 million contribution (Newfoundland & Labrador Executive Council, 2024), the combined annual funding reaches $95 per student, or $0.51 to feed each student per school day—enough to provide only half of a medium-sized apple daily (Health Canada, 2016; Statistics Canada, 2025). In some areas of the province, families also contribute through a pay-what-you-can model, though this support remains limited and inconsistent. These funding gaps highlight the need for transparent funding rationale that reflects the true costs of delivering quality school meals.

In Prince Edward Island (PEI), the provincial government has prioritized a universally accessible pay-what-you-can SFP to accommodate varying financial capacities of families (Office of the Auditor General, 2024). PEI’s combined federal and provincial funding equates to $356 per student annually or $1.93 per student per school day. While this figure is significantly higher than in Newfoundland and Labrador, it remains insufficient to provide universally accessible meals, much less provide free meals, considering an estimated total cost of $5.75 per meal (PEI School Food Program Inc., 2023), and limited reliable funding from parents (Yarr, 2024). These data underscore the scale of investment required to establish comprehensive, universally accessible programs. The dramatically uneven contributions from provincial and territorial governments further raise concerns about whether current federal funding levels are scalable or equitable across larger populations.

These *per capita* estimates offer only a partial scope of the true costs and variability in program needs—and likely overstate the funds available for food alone—because federal funding can also be used for other program expenses, as negotiated by each province/territory. The full costs of implementation include equipment, staffing, maintenance, and facilities, many of which vary and fluctuate across regional market conditions and student populations served. Regional disparities present additional challenges to NSFP implementation. Schools in remote, Indigenous, and low-income communities often face limited access to healthy foods, higher food costs, and logistical barriers compared to urban centres. A standardized funding model that does not fully account for these cost variations and community needs risks exacerbating rather than reducing existing inequities in school food access. To ensure equitable delivery, the NSFP must incorporate regionally responsive funding strategies and consider local capacity constraints to better support diverse school environments.

Despite complex budget planning needs, no federal or provincial efforts or commitments have been made to establish reliable SFP cost estimates. To date, no federal rationale has been published for NSFP and SFIF funding levels. Although federal commitments seem intended to supplement—not replace—provincial efforts, lack of clarity surrounding bilateral agreements raises concerns that provinces may use them to reduce or reallocate their existing school food funding. Protections to safeguard NSFP and SFIF funding are essential for program longevity.

## Nutrition guidelines

Currently, no harmonized guidelines or monitoring mechanisms exist for SFP nutritional quality. The NSFP’s stated goal of “fostering a healthy school food environment, aligning meals, snacks, experiences, and education with Canada’s Food Guide and provincial or territorial nutrition guidelines” (National School Food Policy, 2024) implies that SFPs should adhere to standards across governance levels; however, the policy lacks specificity and clarity for implementation. To reduce ambiguity and promote nutrition equity, standardized nutrition guidelines across provinces and territories are urgently needed to replace the existing patchwork. While a recent provincial report (Office of the Auditor General, 2024) and prior research (Fung et al., 2013; Mullally et al., 2010) indicate that robust, evidence-based guidelines and monitoring improve adherence to school nutrition standards, the absence of concrete NSFP guidelines and monitoring mechanisms creates uncertainty about which guidelines should govern SFPs.

Although Canada’s Food Guide (CFG, 2019) provides qualitative, plate-level, individual guidance on healthy eating, it was not designed for menu planning or evaluation in institutional settings. Implementing CFG 2019 for SFP guidance and monitoring will require the development of practical menu-planning resources and tools for institutional use. It may also have budgetary implications, as CFG 2019 adherence has been associated with higher daily diet costs (Rochefort et al., 2022). Further complicating evaluation, existing provincial nutritional standards are often outdated, and monitoring efforts vary widely (Gaudin et al., 2023). For instance, PEI’s SFP follows a structured, universal model with provincial oversight (Office of the Auditor General, 2024), whereas other provinces rely on community-led programs with limited regulatory oversight (Gaudin et al., 2023).

## Monitoring program impact

The continued absence of clear, evidence-based, up-to-date, and nationally adopted nutrition guidelines—along with feasible, cost-effective oversight mechanisms—risks undermining the NSFP’s effectiveness and limiting the ability to measure returns on investment or drive improvements. Furthermore, without systematic tracking, there is no reliable way to evaluate key performance indicators such as nutritional quality, student participation, equity of access across socioeconomic groups, and stigma. Future frameworks for benchmarking, monitoring, and evaluating the NSFP’s implementation must balance flexibility to meet the diverse monitoring needs of local communities with the need for consistency, transparency, and accountability across programs. Engaging academic support in the development of monitoring and evaluation systems could provide valuable expertise and foster opportunities for collaboration. Establishing national benchmarks and evaluation strategies—through measures such as school-level reporting systems or routine government-led assessments—would enable regional comparisons and support evidence-based policy adjustments.

## Significance

The NSFP represents a pivotal opportunity to transform the health and well-being of children across Canada. By addressing key elements, the NSFP can establish a strong foundation for success. Adequate and sustained funding is essential to ensure equitable access to nutritious meals for all students, regardless of location or circumstance, with investments having far-reaching benefits, including improved child health, academic performance, and local economic support. Further, reliable financial support can promote adherence to evidence-based nutrition standards, bolster provincial and territorial programs, and strengthen local food systems. Clear, standardized guidelines are equally critical to ensure consistent nutritional quality across provinces and create a framework for SFPs grounded in evidence-based nutrition guidance (i.e., CFG, 2019), promoting healthier choices and better health outcomes. Lastly, robust guidelines provide a basis for evaluation and monitoring, enabling continuous program improvement, ensuring accountability, and measuring impacts to justify ongoing investment. Monitoring can also ensure the NSFP’s longevity, addressing questions of funding sufficiency and long-term impacts. This policy presents an opportunity to implement a holistic approach to school food that goes beyond simply feeding students—it has the potential to promote equity across communities, enhance child health, and contribute to local and national economic sustainability.

## Conclusion

A comprehensive and coordinated approach to school food is essential to the success of Canada’s NSFP. While positive, current federal funding may not be sufficient to meet the policy’s objectives, and the lack of robust, standardized guidelines could lead to inconsistent program quality. Moreover, without established guidelines, standards, and evaluation mechanisms, the policy may lack the adaptability to evolve alongside the needs of communities and schools, potentially jeopardizing its longevity and widening inequities. To realize the NSFP’s full potential to deliver meaningful, lasting benefits for students and communities across Canada, we urge policymakers to prioritize sufficient funding, develop clear and evidence-based guidelines, and implement comprehensive monitoring and evaluation systems.

## Data Availability

Not applicable.
